# Does self-medication reduce medical expenditure among the middle-aged and elderly population? A four-wave longitudinal study in China

**DOI:** 10.3389/fpubh.2022.1047710

**Published:** 2023-01-11

**Authors:** Zehao Zheng, Zhanchun Feng, Donglan Zhang, Xiaobo Sun, Dong Dong, Youxi Luo, Da Feng

**Affiliations:** ^1^School of Pharmacy, Tongji Medical College of Huazhong University of Science and Technology, Wuhan, China; ^2^School of Medicine and Health Management, Tongji Medical College of Huazhong University of Science and Technology, Wuhan, China; ^3^Division of Health Services Research, Department of Foundations of Medicine, New York University Long Island School of Medicine, Mineola, NY, United States; ^4^School of Statistics and Mathematics, Zhongnan University of Economics and Law, Wuhan, China; ^5^JC School of Public Health and Primary Care, The Chinese University of Hong Kong, Shatin, NT, Hong Kong SAR, China; ^6^School of Science, Hubei University of Technology, Wuhan, China

**Keywords:** self-medication, medical expenditure, middle-aged and elderly population, longitudinal study, two-part mixed-effect model, China

## Abstract

**Introduction:**

Self-medication has a high prevalence in the middle-aged and elderly population in China. Despite the published evidence demonstrating the economic benefits of self-medication, limited research has addressed the relationship between self-medication and individual medical expenditures, especially within the Chinese population. This study examined the effect of self-medication on individual medical expenditures in China and analyzed the heterogeneity between outpatient and inpatient cases.

**Methods:**

We conducted a panel data analysis using data from four waves of the China Health and Retirement Longitudinal Study (CHARLS). Two-part mixed-effect models were implemented to estimate the effect of self-medication on total outpatient and inpatient expenses and out-of-pocket (OOP) costs, where mixed-effects logit regression was used as the first part, and generalized linear mixed models with log link and gamma distribution was used as the second part.

**Results:**

We identified 72,041 responses representing 24,641 individuals, of which 13,185 responses incurred outpatient expenses and 9,003 responses incurred inpatient costs. Controlling for all covariates, we found that self-medication behaviors were significantly associated with a higher probability of outpatient service utilization (OR = 1.250, 95% CI = 0.179 to 0.269; *P* < 0.001), but displayed no significant association with outpatient expenses. Respondents who had taken self-medication were less likely to use inpatient services (OR = 0.865, 95% CI = −0.201 to −0.089; *P* < 0.001), and their inpatient expenses were significantly reduced by 9.4% (*P* < 0.001). Inpatient OOP costs were significantly reduced by 10.7% (*P* < 0.001), and outpatient OOP costs were significantly increased by 11.3% (*P* < 0.001) among respondents who had self-medicated.

**Conclusions:**

This study allowed us to identify the economic value of self-medication among the middle-aged and elderly population in China. Future work should guide the middle-aged and elderly to take responsible self-medication to reduce their economic burden.

## 1. Introduction

As a global public health issue, self-medication has become part of the healthcare system. The World Health Organization (WHO) defines it as the “selection and use of [herbal or chemical] medicines by individuals to treat self-recognized illnesses or symptoms” (1). The World Self-Medication Industry (WSMI) emphasizes responsible self-medication, describing it as “the practice whereby individuals treat their ailments and conditions with medicines which are approved and available without prescription, and which are safe and effective when used as directed” (2). Self-medication has positive effects on both personal health and healthcare systems; thus, it has attracted widespread attention in recent years (3, 4). With the improvement of the population's health literacy, self-medication has played an increasingly important role in the medical system.

Self-medication has become a common phenomenon that is widely practiced worldwide. As shown by previous studies, at least 43.8% of respondents self-medicated frequently in the United States (5), the prevalence of self-medication was approximately 25 % among older Europeans (6), and the prevalence rate in Spain was 12.7% (7). In developing countries, the prevalence of self-medication was 26.3% in Chile (8) and 76.3% in Brazil (9). Self-medication has a long history and a solid mass foundation in China (10). During the previous 12 months, 74.6% of respondents practiced self-medication (11). Furthermore, 45.4% of respondents would select self-medication if they felt physical discomfort during the 2 weeks preceding the survey, which was higher than the proportion who chose to “see the doctor” (12). Among the middle-aged and elderly people in China, the prevalence of self-medication during the previous month was 45.52% (13). Such a high prevalence has sparked a strong focus on self-medication among scholars.

Self-medication of middle-aged and elderly people shows particularity who seem to have a higher susceptibility to self-medicate (14). It is well established from a variety of studies that the elderly are the largest consumers of medicines in most countries (15, 16). The existing research recognizes that the elderly are one of the population groups with a higher prevalence of self-medication, resulting from a higher prevalence of diseases (17). On the one hand, age-related changes in pharmacokinetics and pharmacodynamics put the middle-aged and elderly at higher risk of medication than other age groups (18). On the other hand, their choices of medication are prone to be influenced by the surrounding environment. A wide variety of pharmaceutical products on the market, and the extensive publicity surrounding these products, often target the middle-aged and elderly (19). In this context, irresponsible self-medication by middle-aged and elderly people frequently occurs, with harmful consequences. All of these make self-medication by the middle-aged and elderly extremely complicated, which deserves major attention.

It is worth noting that self-medication has potential risks if taken inappropriately. Without proper consultation from healthcare professionals, self-medication increases the risk of drug abuse (20), consumption of inappropriate medication (21), and adverse drug events (22). In the meantime, the high proportion of sales of antibiotics without a prescription in China has been reported in the body of literature (23). Self-medication has exacerbated the abuse of antibiotics, which contributes to the development of antibiotic resistance. Despite its negative effects, the advantages of self-medication, especially the economic benefits, have been widely recognized. With the rapid growth of medical expenses, cost containment has gradually become a consensus in the healthcare system (24, 25). Previous research has shown that self-medication plays an active role in decreasing healthcare costs (26). Self-medication reduces the burden on healthcare services caused by minor and trivial ailments, leaving doctors with more energy to deal with patients in need (27). Self-medication reduces the need for clinic visits, thereby enabling cost reductions, which is the major cause of self-medication among patients (4, 28). Time saving is another factor that motivates patients to self-medicate (29, 30). Patients avoid spending travel time to the hospital and waiting in line for medical services at clinics or physicians' offices. Without a doubt, self-medication brings savings in costs and increased productivity (31). Studies have revealed that 45.5% of respondents performed self-medication to save money and 82% did so to save time (32).

Although many studies have explored the economic value of self-medication and affirmed its role in reducing medical expenditures (33–35), most quantitative studies concentrated on the national level or on certain diseases. Much of the research focused on the individual patient has been, up to now, descriptive in nature, and analyses of data about the savings in costs caused by self-medication from the perspective of patients are limited. This study conducted a panel data analysis using data from the 2011, 2013, 2015, and 2018 waves of the China Health and Retirement Longitudinal Study (CHARLS), and examined the effect of self-medication on individual medical expenditures among the middle-aged and elderly population in China. The heterogeneity between outpatient and inpatient cases was also analyzed. Our study quantified the effect of self-medication on savings in the cost of healthcare and its reduced burden on healthcare services. Our work also filled the gaps in the research on the middle-aged and elderly population in China. In the meantime, the CHARLS provided longitudinal data that delivered more accurate estimates.

## 2. Materials and methods

### 2.1. Data source

Our work is a longitudinal study based on data from four waves of the China Health and Retirement Longitudinal Study (CHARLS), namely 2011, 2013, 2015, and 2018. The CHARLS is a national population-based survey that includes assessments of social, economic, and health circumstances of community residents. The CHARLS collects high-quality data through face-to-face interviews with a structured questionnaire. The project, using multistage stratified probability-proportionate-to-size sampling, selected a nationally representative sample of Chinese residents aged 45 years and older. The data were collected from 28 provinces, 150 counties/districts, and 450 villages/urban communities across the country. The total sample size of the CHARLS baseline survey in 2011 was 17,708 individual respondents. Around 70% of the original 2011 sample participated in the follow-up survey throughout the following waves, and the response rate was over 86% (36). Detailed descriptions of the survey design and procedures can be found in in the original study documentation (37).

In this study, we included data from participants who had responded in “Module E: Health Care and Insurance”, and we excluded respondents who had missing values of dependent or independent variables. Respectively, 16,966, 16,946, 19,603, and 18,526 samples were included in Wave 1, 2, 3, and 4. In view of the statistical method used, the final analytical sample was not necessarily a balanced panel. A response from certain individuals might be excluded from our analyses while the rest of the responses from the same individuals might be included (38).

### 2.2. Study variables

#### 2.2.1. Self-medication

In the CHARLS Waves 1, 2, and 3, the question about self-medication was: “How did you treat yourself during the past month?” The answer options included: (1) Consumed over-the-counter modern medicines; (2) Consumed prescription medicines; (3) Consumed traditional herbs or traditional medicines as treatment; (4) Consumed tonics/health supplements; (5) Used healthcare equipment; (6) Others; and (7) None. If the respondent chose consumed over-the-counter modern medicines, prescription medicines, or traditional herbs or traditional medicines as treatment, he was regarded as taking self-medication (13). In Wave 4, three types of medications were combined. The question about self-medication was: “Did you take any purchased medicine during the past month?” The respondents replied “Yes”, which indicated taking self-medication. These two definitions are consistent with the meaning of self-medication defined by the WHO (1).

#### 2.2.2. Dependent variables

In the CHARLS, medical expenditure was self-reported as the total amount paid during 1 month preceding the survey date for outpatients or 1 year preceding the survey date for inpatients. Participants were asked about their outpatient expenditure *via* the following question: “How much did all the visits to medical facilities cost during the last month? (Include self-paid part and reimbursement part)”. These medical facilities were reported by participants as places they had visited in the last 4 weeks for outpatient treatment. The self-paid part was asked separately and defined as outpatient out-of-pocket (OOP) costs. Inpatient expenditure was asked *via* the following question: “What was the medical cost for all the hospitalizations you received during the past year? (Only include fees paid to the hospital, including ward fees but excluding wages paid to a hired nurse, transportation costs, and accommodation costs for yourself or family members).” The self-paid part of inpatient expenditure was defined as inpatient OOP costs.

#### 2.2.3. Independent variables

We used Anderson's behavior model to select independent variables, including predisposing factors, enabling factors, and need factors (39). The classifications of the categorical variables were informed by published evidence. Predisposing factors included age, gender, marital status (40) (married = married with spouse present/cohabitated, and unmarried = married but not living with spouse temporarily for reasons such as work/separated/divorced/widowed/never married), and education status (41) (primary school and below and secondary school and above). Enabling factors included residential area (42) (rural and urban), socioeconomic status quartiles [annual per capita household consumption expenditure (43)], region (east, west, and center), and medical insurance schemes. Need factors consisted of self-rated health (very good, good, fair, poor, and very poor) and number of chronic diseases. Different medical insurance schemes were considered in our study, including Urban employee medical insurance (UEBMI), Urban resident medical insurance (URBMI), New rural cooperative medical insurance (NCMS), and Urban and rural resident medical insurance (URRBMI). Multiple/Other was indicated if the patient was a policyholder or primary beneficiary of more than one type of listed health insurance, or a policyholder or primary beneficiary of any insurance other than UEBMI, URBMI, NCMS, and URRBMI (44). Health behaviors such as smoking (yes and no) and drinking (often, sometimes, and never) were also considered.

### 2.3. Statistical analysis

Generally, medical expenditure is not normally distributed and shows more zero numbers. The two-part model (TPM) has been widely used for analyzing medical expenditure to address the problem of excess zeroes (45). The first part estimated the likelihood of an individual incurring any expenditure by a logit model. The second part was a generalized linear regression estimating medical expenditure among those with positive expenditure. The TPM with random effects could provide a method for analyzing repeated measurement data. Two-part mixed-effect models have been used to analyze longitudinal count data with excess zeros (46).

#### 2.3.1. Part 1 – Selection equation: Mixed-effects logit regression

Selection equation, the first part of the two-part mixed-effect model, considered a binomial distribution – whether any medical expenditures was incurred (y > 0) or not (y = 0) – and implemented a mixed-effects logit regression to estimate the probability of medical service utilization. There is N number of participants, each of whom had multiple interviewed records. The probability function of medical service utilization was defined as:


(1) $$\begin{array}{l} \mathrm{Pr}\left(y_{ij}>0|x_{ij},u_{i}\right)=G\left(x_{ij}\beta_{p1}+u_{i}+\varepsilon_{ij}\right) \end{array}$$

Here, *i* = 1, …, *N* individuals, with the *i* individual having *j* = 1, …, *n*_*i*_ interviewed records. The outcome (*y*_*ij*_) was a binary response, where *y*_*ij*_ > 0 if medical expenditure was positive, and *y*_*ij*_ = 0 if otherwise. The outcome could be influenced by a set of fixed effects *x*_*ij*_ and random effects *u*_*i*_. β_*p*1_ were their associated regression coefficients for the Part 1 model. Considering no random slope, *u*_*i*_ was the random intercept for each individual (47). For the logit regression, this function estimated the probability of (*y*_*ij*_ > 0).

#### 2.3.2. Part 2 – Regression equation: Mixed-effects linear model with log link and gamma distribution

Conditional to any medical expenditure incurred (*y*_*ij*_ > 0), the intensity of medical expenditure could be fitted with a generalized linear mixed model (GLMM) called “regression equation”. The GLMM, an extension of the generalized linear model and mixed linear model, could process hierarchical data, making it suitable for the CHARLS database. Including random effects in the model addressed the problems of correlations, excessive dispersion, and heterogeneity among the data. The GLMM was also insensitive to missing data, which reduced the bias in the study's outcomes caused by data missing from the CHARLS database. The estimated intensity of medical expenditure was defined as:


(2) $$g[E(y_{ij})]=x_{ij}\beta_{p2}+U_{i}+\upsilon_{ij}\;,\;\;\;y_{ij}>0$$

Here, *i* = 1, …, *N* individuals, with the *i* individual having *j* = 1, …, *n*_*i*_ interviewed records with positive expenditure. The outcome (*y*_*ij*_) represented medical expenditure of each individual. β_*p*2_ were the associated regression coefficients for the Part 2 model and *x*_*ij*_ were set as fixed effects. Without considering any random slopes, *U*_*i*_ indicated the random intercept for each individual (47). The generalized linear mixed model was specified as log link and gamma distribution, which are commonly used in econometric analyses of medical expenditure due to their asymptotic properties for non-negative outcomes (48).

### 2.4. Statistical software

All statistical calculations were carried out using the R software (version 4.1.2; R Development Core Team, URL http://www.R-project.org, 2021). Generalized linear mixed models were performed using the library “glmmTMB”. The optimizer was considered to adjust the model.

## 3. Results

### 3.1. Sample characteristics

We identified 72,041 responses representing 24,641 individuals in the unbalanced panel data, of which 13,185 responses incurred outpatient expenses and 9,003 responses incurred inpatient costs. A total of 9,338 eligible individuals had incurred outpatient costs 1 month before the interview, and they generated 1–4 responses among the four waves. Table 1 summarizes the demographics of the included outpatients in different years. The individuals were aged 59.73 years on average and consisted of 41.43% men and 58.57% women. Of these, 8,162 (61.90%) respondents had taken self-medication 1 month before the interview. Women (59.00%) had performed more self-medication, same as the respondents that were married (87.18%), had attended primary school or below (66.93%), or were living in rural areas (74.78%).

**Table 1:** Demographic characteristics of outpatients (*N* = 13,185).

**Groups**		**2011 (*****N =*** **3,115)**		**2013 (*****N =*** **3,526)**		**2015 (*****N =*** **3,613)**		**2018 (*****N =*** **2,931)**	
		***n**,* **%**		***n**,* **%**		***n*** **, %**		***n*** **, %**	
Self-medication	No	1,436	46.10	1,266	35.90	1,393	38.56	928	31.66
	Yes	1,679	53.90	2,260	64.10	2,220	61.44	2,003	68.34
Age	< 45	75	2.41	88	2.50	184	5.09	40	1.36
	45~54	1,006	32.30	1,103	31.28	1,150	31.83	854	29.14
	55~64	1,151	36.95	1,287	36.50	1,189	32.91	937	31.97
	≥65	883	28.35	1,048	29.72	1,090	30.17	1,100	37.53
Gender	Female	1,829	58.72	2,082	59.05	2,094	57.96	1,718	58.61
	Male	1,286	41.28	1,444	40.95	1,519	42.04	1,213	41.39
Educational status	Middle	882	28.31	1,127	31.96	1,264	34.98	1,012	34.53
	Primary	2,233	71.69	2,399	68.04	2,349	65.02	1,919	65.47
Residential area	Rural	2,517	80.80	2,734	77.54	2,607	72.16	2,094	71.44
	Urban	598	19.20	792	22.46	1,006	27.84	837	28.56
Marital status	Married	2,699	86.65	3,060	86.78	3,178	87.96	2,496	85.16
	Unmarried	416	13.35	466	13.22	435	12.04	435	14.84
Region	East	1,016	32.62	1,080	30.63	1,178	32.60	936	31.93
	Mid	1,034	33.19	1,115	31.62	1,123	31.08	907	30.95
	West	1,065	34.19	1,331	37.75	1,312	36.31	1,088	37.12
Self-rated health	Very good	34	1.09	62	1.76	107	2.96	125	4.26
	Good	180	5.78	201	5.70	200	5.54	216	7.37
	Fair	739	23.72	919	26.06	983	27.21	1,321	45.07
	Poor	1,295	41.57	1,445	40.98	1,412	39.08	922	31.46
	Very poor	867	27.83	899	25.50	911	25.21	347	11.84
Number of chronic diseases	0	528	16.95	622	17.64	700	19.37	274	9.35
	1	858	27.54	926	26.26	787	21.78	492	16.79
	≥2	1,729	55.51	1,978	56.10	2,126	58.84	2,165	73.87
Medical insurance scheme	None	154	4.94	120	3.40	302	8.36	68	2.32
	NCMS	2,356	75.63	2,610	74.02	2,341	64.79	1,705	58.17
	UEBMI	289	9.28	415	11.77	386	10.68	320	10.92
	URBMI	121	3.88	171	4.85	132	3.65	112	3.82
	URRBMI	36	1.16	51	1.45	73	2.02	321	10.95
	Multiple/other	159	5.10	159	4.51	379	10.49	405	13.82
Smoke	No	2,360	75.76	2,529	71.72	2,828	78.27	2,340	79.84
	Yes	755	24.24	997	28.28	785	21.73	591	20.16
Drink	No	2,285	73.35	2,541	72.06	2,498	69.14	2,086	71.17
	Often	595	19.10	705	19.99	816	22.59	620	21.15
	Sometimes	235	7.54	280	7.94	299	8.28	225	7.68

Among the four waves, we identified 9,003 responses representing 6,861 individuals that had incurred inpatient costs 1 year before the interview. Table 2 summarizes the demographics of the included inpatients over the 4 years. The respondents were aged 62.66 years on average and consisted of 47.98% men and 52.02% women. Of these, 5,381 (59.77%) respondents had taken self-medication 1 month before the interview.

**Table 2:** Demographic characteristics of inpatients (*N* = 9,003).

**Groups**		**2011 (*****N =*** **1,472)**		**2013 (*****N =*** **2,073)**		**2015 (*****N =*** **2,421)**		**2018 (*****N =*** **3,037)**	
		***n*** **, %**		***n*** **, %**		***n*** **, %**		***n*** **, %**	
Self-medication	No	701	47.62	842	40.62	1,039	42.92	1,040	34.24
	Yes	771	52.38	1,231	59.38	1,382	57.08	1,997	65.76
Age	< 45	20	1.36	30	1.45	76	3.14	24	0.79
	45~54	381	25.88	517	24.94	573	23.67	578	19.03
	55~64	531	36.07	785	37.87	787	32.51	897	29.54
	≥65	540	36.68	741	35.75	985	40.69	1,538	50.64
Gender	Female	754	51.22	1,076	51.91	1,274	52.62	1,579	51.99
	Male	718	48.78	997	48.09	1,147	47.38	1,458	48.01
Educational status	Middle	447	30.37	633	30.54	850	35.11	928	30.56
	Primary	1,025	69.63	1,440	69.46	1,571	64.89	2,109	69.44
Residential area	Rural	1,088	73.91	1,571	75.78	1,665	68.77	2,113	69.58
	Urban	384	26.09	502	24.22	756	31.23	924	30.42
Marital status	Married	1,277	86.75	1,790	86.35	2,097	86.62	2,484	81.79
	Unmarried	195	13.25	283	13.65	324	13.38	553	18.21
Region	East	398	27.04	564	27.21	698	28.83	788	25.95
	Mid	485	32.95	679	32.75	784	32.38	1,022	33.65
	West	589	40.01	830	40.04	939	38.79	1,227	40.40
Self-rated health	Very good	16	1.09	37	1.78	49	2.02	121	3.98
	Good	75	5.10	110	5.31	111	4.58	194	6.39
	Fair	283	19.23	477	23.01	554	22.88	1,177	38.76
	Poor	622	42.26	788	38.01	936	38.66	1,045	34.41
	Very poor	476	32.34	661	31.89	771	31.85	500	16.46
Number of chronic diseases	0	213	14.47	289	13.94	353	14.58	198	6.52
	1	333	22.62	472	22.77	466	19.25	412	13.57
	≥2	926	62.91	1,312	63.29	1,602	66.17	2,427	79.91
Medical insurance scheme	None	59	4.01	58	2.80	193	7.97	46	1.51
	NCMS	1,026	69.70	1,487	71.73	1,480	61.13	1,754	57.75
	UEBMI	206	13.99	289	13.94	311	12.85	386	12.71
	URBMI	73	4.96	95	4.58	122	5.04	126	4.15
	URRBMI	16	1.09	38	1.83	45	1.86	305	10.04
	Multiple/other	92	6.25	106	5.11	270	11.15	420	13.83
Smoke	No	1,112	75.54	1,473	71.06	1,915	79.10	2,417	79.59
	Yes	360	24.46	600	28.94	506	20.90	620	20.41
Drink	No	1,108	75.27	1,546	74.58	1,742	71.95	2,263	74.51
	Often	272	18.48	389	18.77	500	20.65	572	18.83
	Sometimes	92	6.25	138	6.66	179	7.39	202	6.65

### 3.2. Descriptive statistics

**Figure 1:**
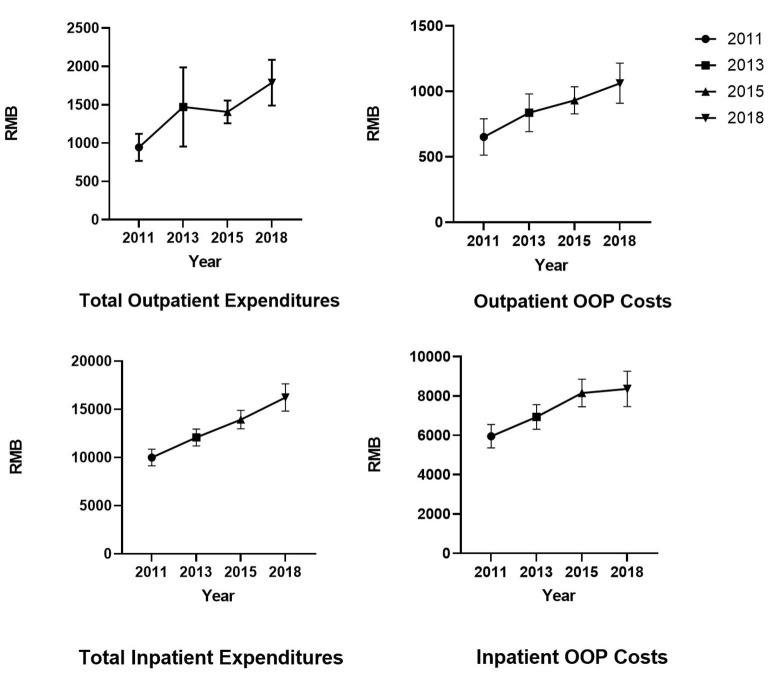
The mean of total medical expenditures and out-of-pocket (OOP) costs.

Figure 1 reports the mean of total medical expenditures and out-of-pocket (OOP) expenditure in different groups. Costs were adjusted to 2018 RMB using the consumer price index. The mean of monthly total outpatient costs in 2011, 2013, 2015, and 2018 was ¥944.61, ¥1,471.71, ¥1,407.68, and ¥1,789.23, respectively. The mean of annual total inpatient costs in 2011, 2013, 2015, and 2018 was ¥9,991.01, ¥12,068.57, ¥13,932.96, and ¥16,227.11, respectively. Although we used CPI to eliminate the effect of inflation, medical expenditure showed an increasing trend over the years. Regarding self-medication, patients who self-medicated had lower inpatient expenditures and OOP costs for inpatient care.

### 3.3. Two-part mixed-effects models

To evaluate the impact of self-medication on medical expenditures, this study applied two-part mixed-effects models. Logit regression was used as the first part and a gamma distribution and log link were performed for the second part. Outpatient expenditures are presented in Table 3 and inpatient expenditures in Table 4.

**Table 3:** Parameter estimates from two-part mixed-effects model on outpatient expenditures.

**Independent variable**	**First part**				**Second part**			
	**Estimate**	**S.E**.	**OR (95%CI)**	***P***	**Estimate**	**S.E**.	**Exp (β)**	***P***
Self-medication	0.223	0.024	1.250 (1.194, 1.309)	< 0.001	0.05	0.029	1.051	0.087
Age	−0.004	0.001	0.996 (0.994, 0.999)	0.003	0.007	0.002	1.007	< 0.001
Gender	−0.094	0.032	0.910 (0.856, 0.968)	0.003	0.262	0.04	1.300	< 0.001
Educational status	0.002	0.026	1.002 (0.952, 1.054)	0.945	−0.016	0.032	0.984	0.62
Residential area	−0.147	0.034	0.863 (0.808, 0.922)	< 0.001	0.235	0.043	1.264	< 0.001
Marital status	0.015	0.035	1.015 (0.947, 1.087)	0.675	−0.126	0.043	0.882	0.004
**Region (Ref: East)**								
Mid	−0.068	0.031	0.934 (0.879, 0.993)	0.028	−0.09	0.04	0.914	0.025
West	0.028	0.031	1.029 (0.969, 1.092)	0.352	−0.124	0.039	0.884	0.002
**Self-rated health (Ref: Fair)**								
Very good	−0.739	0.064	0.477 (0.421, 0.542)	< 0.001	−0.109	0.092	0.897	0.237
Good	−0.493	0.045	0.611 (0.559, 0.667)	< 0.001	−0.076	0.062	0.926	0.217
Poor	0.340	0.027	1.406 (1.333, 1.483)	< 0.001	0.203	0.034	1.225	< 0.001
Very poor	0.705	0.035	2.024 (1.891, 2.166)	< 0.001	0.551	0.041	1.735	< 0.001
Number of chronic diseases	0.206	0.008	1.228 (1.210, 1.247)	< 0.001	0.085	0.009	1.089	< 0.001
Per capita household expense	0.130	0.012	1.138 (1.112, 1.166)	< 0.001	0.231	0.015	1.260	< 0.001
**Medical insurance scheme (Ref: None)**								
NCMS	0.272	0.051	1.312 (1.187, 1.450)	< 0.001	0.003	0.065	1.003	0.969
UEBMI	0.313	0.063	1.368 (1.209, 1.547)	< 0.001	0.306	0.08	1.358	< 0.001
URBMI	0.161	0.076	1.174 (1.011, 1.364)	0.035	0.212	0.096	1.236	0.027
URRBMI	0.248	0.077	1.281 (1.103, 1.489)	0.001	−0.011	0.096	0.989	0.905
Multiple/other	0.393	0.063	1.481 (1.308, 1.677)	< 0.001	0.152	0.079	1.164	0.055
Smoke	−0.271	0.033	0.762 (0.715, 0.813)	< 0.001	−0.277	0.041	0.758	< 0.001
**Drink (Ref: No)**								
Often	−0.151	0.032	0.860 (0.807, 0.916)	< 0.001	−0.291	0.041	0.747	< 0.001
Sometimes	−0.015	0.043	0.985 (0.906, 1.071)	0.724	−0.166	0.052	0.847	0.002
**Year (Ref: 2011)**								
2013	0.121	0.031	1.129 (1.063, 1.198)	< 0.001	0.251	0.036	1.285	< 0.001
2015	−0.068	0.030	0.934 (0.880, 0.992)	0.026	0.342	0.037	1.408	< 0.001
2018	−0.361	0.035	0.697 (0.650, 0.746)	< 0.001	0.495	0.043	1.640	< 0.001

**Table 4:** Parameter estimates from two-part mixed-effects model on inpatient expenditures.

**Independent variable**	**First part**				**Second part**			
	**Estimate**	**S.E**.	**OR (95%CI)**	***P***	**Estimate**	**S.E**.	**Exp (β)**	***P***
Self-medication	−0.145	0.028	0.865 (0.818, 0.915)	< 0.001	−0.098	0.025	0.906	< 0.001
Age	0.027	0.002	1.027 (1.024, 1.030)	< 0.001	0.003	0.001	1.003	0.018
Gender	0.454	0.037	1.574 (1.464, 1.693)	< 0.001	0.345	0.031	1.412	< 0.001
Educational status	0.034	0.032	1.035 (0.973, 1.101)	0.281	0.062	0.028	1.064	0.026
Residential area	−0.008	0.040	0.992 (0.918, 1.072)	0.838	0.174	0.035	1.190	< 0.001
Marital status	−0.001	0.041	0.999 (0.922, 1.082)	0.980	−0.069	0.035	0.933	0.048
**Region (Ref: East)**								
Mid	0.179	0.038	1.196 (1.110, 1.289)	< 0.001	−0.321	0.034	0.725	< 0.001
West	0.374	0.037	1.453 (1.351, 1.564)	< 0.001	−0.394	0.033	0.674	< 0.001
**Self-rated health (Ref: Fair)**								
Very good	−0.665	0.078	0.514 (0.441, 0.599)	< 0.001	−0.03	0.079	0.970	0.703
Good	−0.401	0.056	0.670 (0.600, 0.748)	< 0.001	−0.028	0.056	0.973	0.622
Poor	0.496	0.034	1.642 (1.537, 1.754)	< 0.001	0.157	0.03	1.171	< 0.001
Very poor	1.067	0.041	2.908 (2.683, 3.152)	< 0.001	0.349	0.034	1.418	< 0.001
Number of chronic diseases	0.271	0.009	1.312 (1.289, 1.335)	< 0.001	0.026	0.007	1.026	< 0.001
Per capita household expense	0.269	0.015	1.309 (1.272, 1.347)	< 0.001	0.322	0.012	1.379	< 0.001
**Medical insurance scheme (Ref: None)**								
NCMS	0.462	0.067	1.588 (1.393, 1.810)	< 0.001	−0.027	0.063	0.974	0.669
UEBMI	0.643	0.079	1.902 (1.631, 2.219)	< 0.001	0.312	0.072	1.367	< 0.001
URBMI	0.481	0.093	1.618 (1.347, 1.943)	< 0.001	0.106	0.085	1.112	0.208
URRBMI	0.463	0.092	1.589 (1.326, 1.904)	< 0.001	0.003	0.083	1.003	0.97
Multiple/other	0.653	0.079	1.922 (1.646, 2.244)	< 0.001	0.182	0.072	1.199	0.011
Smoke	−0.462	0.039	0.630 (0.584, 0.680)	< 0.001	−0.306	0.033	0.736	< 0.001
**Drink (Ref: No)**								
Often	−0.423	0.039	0.655 (0.607, 0.707)	< 0.001	−0.236	0.034	0.790	< 0.001
Sometimes	−0.284	0.054	0.753 (0.678, 0.837)	< 0.001	−0.17	0.049	0.844	< 0.001
**Year (Ref: 2011)**								
2013	0.380	0.040	1.462 (1.351, 1.582)	< 0.001	0.21	0.036	1.233	< 0.001
2015	0.337	0.039	1.400 (1.296, 1.513)	< 0.001	0.263	0.036	1.301	< 0.001
2018	0.569	0.042	1.767 (1.626, 1.919)	< 0.001	0.307	0.038	1.359	< 0.001

The estimator showed that self-medication behaviors were significantly associated with a higher probability of outpatient service utilization (OR = 1.250, 95% CI = 0.179 to 0.269; *P* < 0.001), but displayed no significant association with outpatient expenses (*P* = 0.087). Respondents who took self-medication were less likely to use inpatient services (OR = 0.865, 95% CI = −0.201 to −0.089; *P* < 0.001), and their inpatient expenses were significantly reduced by 9.4% (*P* < 0.001). The second part indicated a significant increase in healthcare expenditures with increasing age (*P* < 0.001; *P* = 0.018). Women had lower total medical expenditures compared with men (*P* < 0.001). Respondents living in non-rural areas were significantly associated with lower medical costs (*P* < 0.001), same as those living in central and western regions (*P* < 0.001, *P* < 0.001; *P* = 0.025, *P* = 0.002). Respondents who self-reported with “poor” and “very poor” health levels were found to have significantly higher medical expenditures (*P* < 0.001). With the increase in the number of chronic diseases, a significant increase in medical expense was also observed (*P* < 0.001). Regarding socioeconomic status, respondents with higher per capita household expenditure were significantly associated with higher medical costs (*P* < 0.001).

Table 5 shows that annual inpatient OOP costs were significantly reduced by 10.7% (*P* < 0.001) and monthly outpatient OOP costs were significantly increased by 11.3% (*P* < 0.001) in respondents who had taken self-medication. Nevertheless, the impact of medical insurance schemes on total costs and OOP costs showed great heterogeneity. Compared to the uninsured cases, all schemes increased total medical expenditure whether outpatient or inpatient, but statistically significant differences were only found in UEBMI (*P*_outpatient_ < 0.001; *P*_inpatient_ < 0.001) and Multiple/Other insurances (*P*_inpatient_ = 0.011). On the other hand, none of the schemes had a significant effect on outpatient out-of-pocket expenditures. In particular, the percentage savings for inpatient out-of-pocket expenditures were 21.0% (*P* = 0.011) in UEBMI, 16.6% (*P* = 0.026) in NCMS, and 22.8% (*P* = 0.006) in Multiple/Other insurances.

**Table 5:** Parameter estimates from regression equations on OOP costs.

**Independent variable**	**Outpatient OOP**				**Inpatient OOP**			
	**Estimate**	**S.E**.	**Exp (β)**	***P***	**Estimate**	**S.E**.	**Exp (β)**	***P***
Self-medication	0.107	0.031	1.113	< 0.001	−0.113	0.032	0.893	< 0.001
**Medical insurance scheme (Ref: None)**								
NCMS	−0.065	0.069	0.937	0.343	−0.181	0.082	0.834	0.026
UEBMI	−0.077	0.085	0.926	0.369	−0.236	0.093	0.790	0.011
URBMI	0.179	0.102	1.196	0.078	−0.081	0.108	0.922	0.454
URRBMI	−0.153	0.102	0.858	0.134	−0.139	0.108	0.870	0.197
Multiple/other	−0.142	0.084	0.868	0.092	−0.259	0.094	0.772	0.006

## 4. Discussion

This study was a longitudinal data analysis to investigate the impact of self-medication on the medical expenditures of middle-aged and elderly people in China. In our study, we tried to adopt an empirical approach to estimate the net effect of self-medication behavior on individual patient costs with four waves of data, and explored the heterogeneity of the effect on outpatient, inpatient, total costs, and out-of-pocket costs.

Prior studies showed that cost saving is the main reason for choosing self-medication (49). Additionally, the economic value of self-medication has been fully documented. The Consumer Healthcare Products Association (CHPA) indicated that self-medication reduced the frequency of doctor visits. Without self-medication and OTC (Over-the-Counter) medicines, 90% of consumers would have gone to the doctor instead (50). Each dollar spent on OTC medicines saves the U.S. healthcare system more than USD 7(33), and the availability of self-care medicines generates cost savings of more than USD 146 billion for the healthcare system annually in the US (51). The Association of the European Self-Medication Industry (AESGP) also reported that 1.2 billion minor health issues were self-managed every year with self-care products (35), with responsible self -medication accounting for a large proportion. This condition saved the healthcare systems and national economies EUR 34 billion (35). In Australia, the predicted value of switching 11 categories of current prescription medicines to OTC to promote self-medication resulted in savings of a further $1.1 billion for the healthcare system and almost $730 million for Medicare (34).

In terms of inpatient expenditures, comparable results were obtained in our study. We found that respondents who had self-medicated were less likely to use inpatient services, and their inpatient expenses were significantly reduced by 9.4%. Previous research showed that patients in pain who self-medicated with non-prescription medication were associated with significantly fewer hospitalizations experiences (52). Patients with hemophilia who took self-medication without prior consultation with a physician had an 89% reduction in the number of days they were hospitalized (53). To sum up, our results were consistent with fewer hospitalizations and shorter hospital stays. Furthermore, self-medication behaviors were associated with lower inpatient costs.

Although most studies reported that self-medication can reduce outpatient service utilization (50, 54, 55), we drew the unanticipated conclusion. The estimator indicated that self-medication behaviors were significantly associated with a higher probability of outpatient service utilization but showed no significant association with outpatient expenses. In general, self-medication serves as an alternative to absorb the demand of outpatient services. Patients choose self-medication to avoid visits to medical facilities because of various reasons, but it shows different characteristics among different age groups. A large proportion of the younger group performed self-medication because they felt the disease was too mild to require medical service (56). As regards older groups, the major diseases that led to self-treatment were recurrent and related to aging. Such diseases can often be well controlled by regular over-the-counter medication (57). Variance among groups provided a clue to explaining our unanticipated findings.

One possible explanation for the difference between outpatient and inpatient cases among the middle-aged and elderly population might be health awareness and health literacy. Engaging in self-care activities, such as self-medication, is generally considered as a consequence of health condition awareness (58). Respondents who self-medicated tended to be more health-conscious and more concerned about their own health status. This condition provided them with an advantage in comprehending the importance and methods of early disease detection and treatment (59). Respondents who had taken self-medication implemented better interventions in the early stages of the disease, which reduced the risk of chronic disease progression (60, 61). Possible consequences were an increase in outpatient utilization, due to early interventions, such as disease screening and return visits, and a decrease in inpatient utilization because of the effective control of chronic diseases. Despite these interesting results, the mechanism related to the heterogeneity between outpatient and inpatient cases remains speculative, which is limited by secondary data. Therefore, caution should be warranted in extrapolating the results to all patients. Further studies are required to provide more precise explanations by employing the prospective study design.

As far as the OOP costs were concerned, inpatient respondents showed similar results with total medical expenditure. Unexpectedly, outpatient OOP costs were significantly increased among respondents who had self-medicated. The results reminded us of the determinants of self-medicating behaviors. In developing countries, the main reasoning behind people self-medicating was a simple sign and symptom of disease (30), which was often associated with primary care utilization. The substitution relationship between self-medication and primary health care utilization provided a convenient approach to minor illnesses or health problems. However, when serious health problems arose, respondents were forced to turn to higher-tier hospitals. This meant the outpatient service utilization of respondents who had taken self-medication was likely inclined to non-primary care providers because of the substitution relationship. In China, visits to non-primary care providers showed higher patient cost-sharing, a policy that encouraged patients to seek primary care (62). Lower reimbursement rates resulted in higher out-of-pocket outpatient costs for the group we focused on.

Another significant aspect that deserved attention was medical insurance. In China, the government has developed a universal medical insurance system. The basic insurance system offered by the Chinese government consists of three schemes: UEBMI, which provides health insurance to formal-sector urban employees and retirees; URBMI, which aims to insure the urban residents not covered by the UEBMI, such as students and the self-employed; and NCMS, which is a voluntary medical insurance scheme targeting rural residents (44). In 2016, the State Council announced that it would integrate the NCMS and URBMI into URBMI (Urban and Rural Resident Basic Medical Insurance) to integrate fragmented medical insurance schemes and reduce inequity (63). We found that all schemes were significantly associated with a higher probability of medical service utilization, whether outpatient or inpatient. Medical insurances provided better financial protection for respondents, which lowered the barriers for individuals to seek healthcare services (64, 65). Thus, healthcare utilization by the insured increased accordingly (66), which was more similar to our results. In terms of out-of-pocket costs, different schemes reflected the heterogeneity. Almost all schemes played a role in reducing OOP costs, but they were not significant in outpatient cases. Three schemes were significantly associated with lower inpatient OOP costs, where the percentage savings for inpatient out-of-pocket expenditures were 21.0% in UEBMI, 16.6% in NCMS, and 22.8% in multiple/other insurances. The deficient reimbursement levels of medical insurance on outpatient care has been a problem in China's social health insurance schemes (44, 67). Meanwhile, the fragmentized social health insurance schemes operate in isolation, creating inequalities in the system across insurance programs (68). This situation was reflected in our findings: (1) UEBMI individuals provided the most generous benefit packages (65, 69); (2) A certain gap remains between the actual and expected goals of URRBMI (70), which was integrated from NCMS and URBMI; (3) Private health insurance (PHI) was an important supplement to the basic health insurance schemes (71).

Our research affirmed the economic value of self-medication among the middle-aged and elderly population in China. Responsible self-medication deserves to be promoted because it delivers significant benefits for both patients and healthcare systems. Given the risks of self-medication (72), reasonable guidance for residents is necessary in the future. Systematic public educational measures, such as public lectures, television, and online social networks, are necessary to reduce the harmful effects of improper self-medication. Regulations on drug instructions should be strengthened by relevant departments to provide accurate and understandable information on rational drug use.

At the same time, further studies are needed to evaluate the impact of self-medication on medical expenditure across different groups, such as children, women, and medical students.

## 5. Limitations

This study was limited in several ways. First, we were unable to judge the rationality of self-medication from the questionnaires that did not contain more details on the subjects' self-medication behavior. Although self-medication would cause adverse outcomes if taken inappropriately. Second, some of the variables were based on self-reporting and might be subject to recall bias, especially regarding medical expenditure. Medical expenditure has placed a significant cost burden on Chinese middle-aged and elderly people, as well as an enormous psychological burden. Therefore, the medical expenditure may have been over-reported to a certain extent (73). Finally, we excluded respondents that had utilized medical services but did not provide specific medical expenditure, which would bias the estimate of the probability of medical service utilization in the selection equations.

## Data availability statement

Publicly available datasets were analyzed in this study. This data can be found here: https://opendata.pku.edu.cn/dataverse/CHARLS.

## Author contributions

ZF and XS contributed to the conception and design of the study. ZZ performed the statistical analysis and wrote the first draft of the manuscript. DF and DZ wrote sections of the manuscript. DD and YL provided constructive suggestions in revising the manuscript. All authors contributed to the manuscript revision, read, and approved the submitted version.
